# Lactate in the Regulation of Tumor Microenvironment and Therapeutic Approaches

**DOI:** 10.3389/fonc.2019.01143

**Published:** 2019-11-01

**Authors:** Karen G. de la Cruz-López, Leonardo Josué Castro-Muñoz, Diego O. Reyes-Hernández, Alejandro García-Carrancá, Joaquín Manzo-Merino

**Affiliations:** ^1^Programa de Doctorado en Ciencias Biomédicas, Instituto de Investigaciones Biomédicas, Universidad Nacional Autónoma de México, Ciudad Universitaria, Mexico City, Mexico; ^2^Unidad de Investigación Biomédica en Cáncer, Instituto Nacional de Cancerología, México/Instituto de Investigaciones Biomédicas, Universidad Nacional Autónoma de México, Mexico City, Mexico; ^3^Laboratory of Virus and Cancer, Subdirección de Investigación Básica, Instituto Nacional de Cancerología, Mexico City, Mexico; ^4^Programa de Maestría y Doctorado en Ciencias Médicas, Odontológicas y de la Salud, Maestría en Investigación Clínica Experimental, Universidad Nacional Autónoma de Mexico, Mexico City, Mexico; ^5^Biological Cancer Causing Agents Group, Instituto Nacional de Cancerología, Mexico City, Mexico; ^6^Cátedras CONACyT-Instituto Nacional de Cancerología, Mexico City, Mexico

**Keywords:** lactate, acidification, tumor microenvironment (TME), therapy, immune response

## Abstract

Tumor cells must generate sufficient ATP and biosynthetic precursors in order to maintain cell proliferation requirements. Otto Warburg showed that tumor cells uptake high amounts of glucose producing large volumes of lactate even in the presence of oxygen, this process is known as “Warburg effect or aerobic glycolysis.” As a consequence of such amounts of lactate there is an acidification of the extracellular pH in tumor microenvironment, ranging between 6.0 and 6.5. This acidosis favors processes such as metastasis, angiogenesis and more importantly, immunosuppression, which has been associated to a worse clinical prognosis. Thus, lactate should be thought as an important oncometabolite in the metabolic reprogramming of cancer. In this review, we summarized the role of lactate in regulating metabolic microenvironment of cancer and discuss its relevance in the up-regulation of the enzymes lactate dehydrogenase (LDH) and monocarboxilate transporters (MCTs) in tumors. The goal of this review is to expose that lactate is not only a secondary product of cellular metabolic waste of tumor cells, but also a key molecule involved in carcinogenesis as well as in tumor immune evasion. Finally, the possible targeting of lactate production in cancer treatment is discussed.

## Introduction

Cellular transformation involves the deregulated control of cell proliferation, resistance to cell death, immune evasion and circumvention of growth suppressor activities, which finally allow cancer establishment (1). Additionally, it has been observed that tumor cells have the remarkable ability to adjust their energetic metabolism as part of their mechanisms for tumor survival, this feature is now recognized as a hallmark of cancer (2). The increased metabolic rate in several neoplasms, was first studied by Otto Warburg in 1926 demonstrating that tumor cells uptake high amounts of glucose as a primary energy source, producing excessive amounts of lactate, even in the presence of oxygen (3). In 1972, Efraim Racker named such effect as the “Warburg Effect,” also known as “aerobic glycolysis” (4). Initially, it was proposed that the driving event of the enhanced glycolysis in tumor cells was caused by an irreversible damage of the mitochondrial function. Although defects in mitochondria function have been shown in some types of cancer (5), this process alone cannot explain the metabolic preference of tumor cells.

The Warburg phenotype is present in several neoplasms including breast, colon, cervical and liver cancer (6–9). The increased glucose uptake and metabolism by neoplastic cells represents the basis for tumor detection using positron emission tomography (PET); PET imaging uses a radioisotope-labeled glucose tracer, ^18^F-fluorodeoxyglucose (^18^F-FDG), to identify areas of high glucose uptake/metabolism in the body. After ^18^F-FDG distribution, the radionuclide is transported into the cells by glucose transporters, and consequently phosphorylated by the hexokinase to produce ^18^F-FDG-6-phosphate (^18^F-FDG-6-p). Once inside the cell, the ^18^F-FDG-6-p accumulates in the cytoplasm since this molecule cannot be further metabolized through the glycolytic pathway because it lacks the necessary 2'hydroxyl group (10). Additionally, due to its highly polar nature the ^18^F-FDG-6-p is trapped inside the cell, thus the accumulated amounts of ^18^F-FDG-6-p are used to identify the presence of solid tumors as well as the effectiveness of treatments (10).

The Warburg effect involves the alteration of metabolic enzymes, including hexokinase 2 (HK2), pyruvate kinase type M2 (PKM2), glucose transporter 1 (GLUT1), lactate dehydrogenase (LDH) and lactate transporters (monocarboxilate transporters [MCTs]) (11–14). Importantly, the Warburg phenotype has been associated, not only with an increased obtention of energy but also with the activation of numerous transcription factors, such as c-Myc, NF-κB, and Hypoxia-Inducible Factor 1-α (HIF 1-α) (15–17). These transcription factors can regulate the expression of metabolic enzymes resulting in the deregulated conversion of glucose to lactate (18) then promoting a “tumor lactagenesis” state (19).

Glycolysis is by far less efficient than oxidative phosphorylation for ATP production, and for this reason cancer cells increase their glucose uptake and glycolytic rate. The high utilization of glucose by cancer cells results in the accumulation of extracellular lactate affecting a number of cell types within the tumor microenvironment (TME), composed by a variety of different cell types such as endothelial cells, cancer-associated fibroblasts (CAFs), immune cells and non-cancer stroma cells (20).

**Figure 1:**
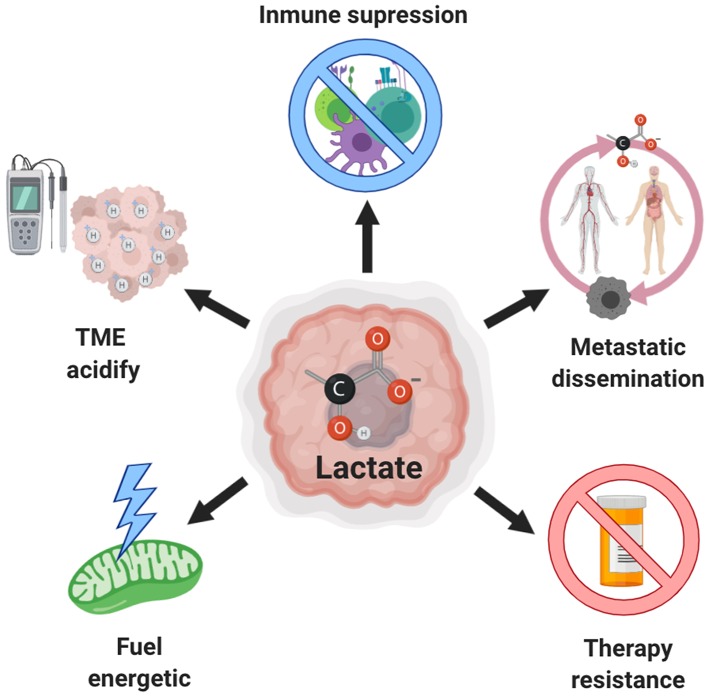
Role of lactate in cancer. Excessive production of lactate by both, tumor and stromal cells, is associated with increased aggressiveness due to the extracellular acidification that also induces invasion and metastasis, inhibition of the antitumor immune response and resistance to therapy. Moreover, this lactate can be used as an alternative source of fuel by tumor cells.

For a long time, lactate was only recognized as a “metabolic waste product” derived of aerobic glycolysis, however, it has now been firmly demonstrated that lactate can be incorporated into the tricarboxylic acid (TCA) cycle and be a source of energy, and even act as an oncometabolite with signaling properties. In this review we describe the role of lactate in tumor progression, highlighting its ability to promote invasion and metastasis. We also show the role of lactate as a metabolic fuel for tumor cells, as well as its participation in drug resistance (Figure 1). The importance of the suppressive acidic tumor microenvironment induced by lactate is also presented. Finally, we discuss the possible targeting of lactate production as a novel therapeutic approach.

## Lactate Metabolism Both in Normal Physiology and Cancer

Lactate (2-hydroxypropanoic acid) is a hydroxycarboxylic acid that may exist in the human body as two stereoisomers, D-lactate and L-lactate; the latter is the predominant physiological enantiomer of lactate (21). D-lactate is also present but generally accounts only for 1–5% of L-lactate concentration (22), in this review we only focus in L-lactate, designed as lactate. The pKa of the lactate/lactic acid pair is 3.8 at physiological pH, the lactic acid dissociates immediately into lactate (base form) and hydrogen (H^+^) (22, 23). Under physiological conditions, lactate is used as a fuel source by the heart, brain and skeletal muscle (24); it can also be converted into glucose in the liver by the Cori cycle, serving as an alternate source of energy (25). Also, lactate acts as an inter-organ carbon shuttle, supplying both aerobic metabolism and gluconeogenesis pathways (26), in fact, now this is identified as a “lactate shuttle theory,” describing that lactate, under fully aerobic conditions can transcend compartment barriers and shuttle occur within and among cells, tissues and organs (27–29), interestingly, this phenomenon is also observed in cancer, and will be described in this review.

The physiological concentration of lactate, in blood and healthy tissues is about 1.5–3 mM (30), but in cancer tissues it can be present in up to 10–30 mM concentrations (31). Table 1 summarizes the amounts of lactate in different neoplasms.

**Table 1:** Lactate quantification in tumors and their association with metastatic spread.

**Cancer type**	**Sample**	**Lactate concentration**	**Method**	**References**
Head and neck cancer	Cryobiopsies from head and neck tumors, either with metastatic spread or without	With metastatic spread: 12.3 ± 3.3 μmol/g	Quantitative bioluminescence imaging	(32)
		Without metastatic spread: 4.7 ± 1.5 μmol/g		
Head and neck cancer	Cryobiopsies from tumors from the head and neck	With metastatic spread: 19.9 μmol/g	Quantitative bioluminescence imaging	(31)
		Without metastatic spread: 7.1 μmol/g		
Cervical cancer	Cryobiopsies at first diagnosis	With metastatic spread: 10.0 ± 2.9 μmol/g	Quantitative bioluminescence imaging	(30)
		Without metastatic spread: 6.3 ± 2.8 μmol/g		
Colorectal cancer	Cryobiopsies from primary rectal adenocarcinoma	With metastatic spread: 13.4 ± 3.8 μmol/g	Quantitative imaging bioluminescence	(33)
		Without metastatic spread: 6.9 μmol/g		
Breast cancer	Cryobiopsies from locally advanced breast cancer	Median concentration range of 0.6–8.0 μmol/g	Quantitative imaging bioluminescence	(34)
Metastatic non-small cell lung cancer	Venous and arterial blood sample	Median maximal levels was 1.8 ± 2.2 mmol/L	Enzymatic method	(35)
Human astrocytomas	Cyst content	With metastatic spread: 12.35 mmol/L	Enzymatic method	(36)
		Without metastatic spread: 8.28 mmol/L		
Head and neck squamous carcinoma	Xenograft in nude mice	More radioresistant tumor ranged 7.3–25.9 μmol/g	Quantitative imaging bioluminescence	(37)
Head and neck squamous, melanoma, rectum carcinoma and adenocarcinoma	Xenograft in nude mice	Median concentration in central areas: 7 μmol/g	Quantitative imaging bioluminescence	(38)
		Median concentration in the periphery region: 0.5 μmol/g		

Glucose is the major source of lactate production in most solid tumors (39). This molecule is an essential metabolic energy source for all living organisms and the structural precursor for cellular biosynthesis of proteins, lipids, and nucleic acids with ATP generation being the essential metabolic process for energy supply to the cells. Mammalian cells generate their ATP through glycolysis in the cytoplasm (2 ATP per glucose molecule) and oxidative phosphorylation (OXPHOS) in the mitochondria (32–34 ATP per glucose molecule). Normal cells (except erythrocytes and skeletal muscle cells during high intensity exercise) depend on OXPHOS for ATP production from glucose; on the contrary, cancer cells obtain their ATP by glycolysis and the final conversion of glucose to lactate (40).

Additionally, it has been demonstrated that glutamine may contribute to a small amount of lactate formation in tumor cells (41). Glutamine comprise the most abundant amino acid in blood circulation (about 500 μM), representing more than 20% of the free amino acid pool in blood and 40% in muscle (42). It was demonstrated that tumor cells require at least 10 times as much glutamine as any other amino acid in culture (43). In the mitochondrion, glutamine is deaminated to glutamate by glutaminase (GLS), later in other deamination reaction, α-ketoglutarate is generated by the enzyme glutamate dehydrogenase (GDH) finally incorporated into TCA cycle to generate malate by fumarase enzyme since α-ketoglutarate is the major anaplerotic source for TCA cycle (44). Malate is exported to the cytosol where is it converted to pyruvate by the malic enzyme which is finally converted to lactate by LDHA (41).

## Lactate as a Fuel Source for Cancer Cells

Despite lactate was first recognized only as a waste product of anaerobic cell metabolism, it is now known that lactate is used continuously as a fuel by diverse cells under complete aerobic conditions (29). Currently it is known that, certain cancer cells may also actively use OXPHOS or a combination of OXPHOS and glycolysis for ATP production (45, 46). Interestingly, using high-resolution mass spectrometry, it was shown that ^13^C-lactate resides inside the mitochondria and can be used as a carbon source to synthesize lipids by cervical cancer and human lung cancer cells (47). Furthermore, it was revealed that LDHB is localized to the inner mitochondrial membrane and was associated with the regulation of the mitochondrial respiration using transmission electron microscopy (TEM) with gold-labeled lactate dehydrogenase B (LDHB) (47). Due to these results, it has been suggested that lactate is oxidized to pyruvate in the mitochondria by LDHB. However, it remains unknown how lactate enters inside the mitochondria in this cells, but it has been proposed that mitochondrial lactate import may be mediated by the monocarboxylate transporter (MCT) as in muscle and neuron cells (48, 49). Another study analyzing [3-^13^C]-lactate metabolism *in vitro* and *in vivo* using nuclear magnetic resonance, indicated that lactate could be transported into and being oxidized by cancer cells (34, 50). Cancer cells are avid consumers of glucose, however, intratumoral levels of glucose are usually exceedingly low (51). Under these circumstances of low glucose, tumor cells uptake and oxidize lactate (52, 53). For instance, breast cancer derived-cells grown in different concentrations of glucose, produce high lactate levels, but switched from net lactate producer to consumers when glucose was limiting (54). Moreover by isotopomer analysis using (U-^13^C)-labeled lactate, it was determined that under conditions of glucose deprivation, over 50% of the total cellular pool of TCA cycle intermediates were derived from lactate (54). Whereas it was shown that lactate can serve as a fuel source when glucose is limited, a disagreement remains in the field as to whether it enters into the TCA cycle directly or if it must first be converted to glucose through gluconeogenesis (55). Further studies are required to decipher its role in cancer, to specifically elucidate what metabolic pathway is preferred and if it is dependent on the tumor metabolism.

Regarding the participation of lactate in the synthesis of TCA cycle intermediaries, Hui et al. (52) used three genetically modified mice cancer models, two for lung cancer and one for pancreas cancer, all under fasting conditions, showing that circulating lactate contributes to the generation of TCA cycle intermediaries. This contribution was higher than of glucose in the two lung cancer mouse models. Using intravenous infusions of ^13^C-labeled nutrients, Faubert et al. (56) showed that the circulatory turnover flux of lactate is the highest of all metabolites and exceeds that of glucose in human lung tumors. Recently, Bok et al. (57) showed that ^13^C-pyruvate is mainly directed to lactate production, associated with tumor progression and metastases.

Although it was shown that glutamine generates lactate in human glioma cells (41), it has been also shown that high amounts of lactate promotes glutamine uptake in SiHa and HeLa cells and consequently induces the glutaminolysis pathway. This increase in the intake and metabolism of glutamine was due to the stabilization of HIF 1-α by lactate. HIF 1-α then transactivates c-MYC proto-oncogene in a pathway that mimics a response to hypoxia. c-MYC is one of the main regulators of glutaminolysis and is also overexpressed in the vast majority of tumors (58). Lactate-induced c-MYC activation triggers the expression of the glutamine transporter ASCT2 and glutaminase 1 (GLS1), both resulting in improved glutamine uptake and catabolism (59).

These findings highlight the use of lactate in the generation of TCA cycle intermediaries and its role as a regulatory molecule of glutamine incorporation and metabolism, to finally serve as a source of energy in cancer cells. Also supports the importance of the mitochondrial function in cancer development.

## Lactate Synthesis: Role of LDHA in Cancer

The inter-conversion between pyruvate and lactate is mediate by the nicotinamide adenine dinucleotide (NAD^+^) oxidoreductase LDH enzyme. This is a tetrameric enzyme composed of M and H protein subunits that are encoded by the LDHA and LDHB genes, respectively (60). The two subunits can then combine and form five homo or hetero tetramers in human tissues: LDH-1 (4H), LDH-2 (3H1M), LDH-3 (2H2M), LDH-4 (1H3M), and LDH-5 (4M). LDH5, also known as LDHA, is the predominant isoform found in skeletal muscle. In contrast, LDH1 also known as LDHB, is the predominant isoform found in heart muscle (61). LDHA preferentially reduces pyruvate to lactate, while LDHB supports conversion of lactate to pyruvate in cells that utilize lactate as a nutrient source for oxidative metabolism or gluconeogenesis (62). Pyruvate is reduced to produce lactate while NADH is oxidized to NAD^+^ in a thermodynamically favored reaction. In the opposite direction, lactate is oxidized to form pyruvate, while NAD^+^ is reduced to NADH (63).

### LDHA Expression in Tumors

Several reports indicate that LDHA expression and its activity is increased in numerous types of tumors and is associated with lower event-free survival rate and with resistance to chemotherapy treatment. For instance, high LDHA levels in serum could be a negative prognostic biomarker in osteosarcoma, pancreatic cancer, and lung adenocarcinoma (64–67). On the other hand, knocking down the expression of LDHA in lung adenocarcinoma cells inhibits the proliferation, invasion, migration and colony formation (67). In human lymphoma and pancreatic cancer, knocking down the expression of LDHA by siRNA reduces ATP levels and induces significant oxidative stress and cell death in human lymphoma and pancreatic cancer xenografts in mice (68).

The ability to monitor when a disease arises, how it progresses and to evaluate the result of treatment through non-invasive techniques is the most desirable goal in clinical setting. Non-invasive sampling is the most useful and valuable alternative because no stress is generated in the oncological patient. LDHA determination in saliva sample has been proposed for detection and monitoring of oral squamous cell carcinoma (OSCC), since the major source of salivary LDHA are the oral epithelium-shedding cells. Any pathological changes in the oral epithelium should be reflected diagnostically in the saliva, and the aggressiveness of different histological grades of OSCC could be assessed (69). Thus, the LDHA levels could be an excellent diagnostic marker. In this regard, a positive correlation between the LDHA expression and the histopathological grading was found in saliva samples from patients with OSCC (70). The expression of salivary LDHA in patients with OSCC was significantly higher than that of healthy individuals. Importantly, the levels of salivary LDHA in patients with squamous cell carcinoma of the tongue and lower oral cavity were significantly higher than other patients affected with squamous cell carcinoma in other parts of the head and neck (71).

### Phosphorylation of LDHA and Its Role in Cancer

It was demonstrated that LDHA is phosphorylated at two specific tyrosine sites, tyrosine 10 (Y10), and tyrosine 83 (Y83). Phosphorylation in Y10 increases LDHA activity by enhancing the active tetrameric LDHA conformation, which induces the binding of NADH and promotes Warburg effect in human head and neck squamous cell carcinoma (HNSCC), lung cancer, breast cancer and prostate cancer cells (72). Interestingly, it was demonstrated that high levels of phosphorylated LDHA in human prostate cancer tissues were associated with short recurrence and poor survival times in patients (73). The tyrosine kinases involved in the Y10 phosphorylation of LDHA are HER2, the avian sarcoma viral oncogene v-src homolog (Src) and the Fibroblast growth factor receptor 1 (FGFR1), this phosphorylation promotes the Warburg effect and pro-invasive and pro-metastatic potential of cancer cells (73, 74). Recently, it was identified that cyclin G2 could directly interact with LDHA and negatively regulate the phosphorylation of Y10 in LDHA, although the mechanism by which cyclin G2 reduce the Y10 phosphorylation remains unknown, this interaction inhibits the Warburg effect and tumor progression in glioma (75). Taken together the phosphorylated-induced activation of LDHA provides other mechanism used by tumor cells in order to establish a malignant phenotype. However, there are very few studies on this topic, so it is important to investigate whether post-translational modifications such as phosphorylation in metabolic enzymes such as LDHA are part of the broad mechanism by which tumorigenesis is promoted by associating signaling and metabolism pathways.

## Non-canonical Functions of LDH in Cancer

Metabolic enzymes exhibit “promiscuous” catalytic activities (76). In addition to the above-described canonical functions of LDH, it has recently been demonstrated that LDHA exhibits non-canonical roles, which are also involved in tumor progression.

Based on this, Intlekonfer et al. (77) observed that LDHA produces the oncometabolite L-2-hydroxyglutarate (L-2HG) under hypoxic conditions in glioblastoma, via alternative substrate usage and additional contributions from malate dehydrogenase 1 and 2 (MDH 1/2). The authors also demonstrated that during hypoxia, the resulting increase in L-2HG is necessary and sufficient for the induction of increased methylation of histone repressive marks such as histone 3 lysine 9 (H3K9me3). Later, the same research group also demonstrated that the L-2HG produced by LDHA is favored in an acidic environment and promotes the HIF 1-α stabilization under normoxia conditions (78). HIF 1-α is associated with metabolic regulation, specifically with tumor lactagenesis, because of the induction of the expression of LDHA, MCT4 and the membrane-bound carbonic anhydrase IX (CAIX), and in this way regulates the tumor acid environment and tumor progression (79, 80). Recently, it has been reported that LDHA translocates to the nucleus, induced by reactive oxygen species (ROS) in cervical cancer. Once in the nucleus, LDHA in its dimeric form produces the antioxidant metabolite α-hydroxybutyrate (α-HB). α-HB induces H3K79 hypermethylation through the interaction between methyl-transferase DOT1L and LDHA, demonstrating that LDHA nuclear translocation appears to be essential for maintaining redox balance and sustaining cell proliferation through epigenetic regulation (81).

This promiscuous enzymatic activity of LDHA might represent a metabolic response to multiple environmental stimuli including hypoxia and acidosis, conditions frequently found in tumor microenvironment of aggressive tumors. Future investigations will be directed at elucidating the role as well as how deregulation L-2HG and α-HB by LDHA might contribute to oncogenesis.

## Lactate Transport: Role of MCTs in Cancer

Eukaryotic cells require the efflux of lactate and H^+^ to the extracellular space to prevent intracellular acidification and sustain continuously high rates of glycolysis, since the accumulation of cytosolic lactate reduces the glycolytic rate via inhibition of the rate-limiting enzyme fosfofructokinase-1 (PFK-1) (82). Lactate itself cannot cross the plasma membrane by free diffusion. Hence, it requires a specific transport mechanism provided by proton-like MCTs (83, 84) It was identified that lactate shuttle is driven by a concentration and pH gradient or by the cellular redox state in rat skeletal muscle (28).

MCTs belong to the family of solute carrier (SLC) transporters, composed by 52 families of the membrane transport proteins; in particular, the SLC16 family encodes 14 MCTs isoforms and plays a significant role in the absorption, tissue distribution and clearance of both, endogenous and exogenous compounds (83). MCTs 1–4 are known lactate transporters, but they can carry other monocarboxylates such as pyruvate and the ketone bodies such as acetoacetate, β-hydroxybutyrate and acetate (85). Two proteins, basigin (CD147) and embigin (gp70), have been identified as important chaperone proteins implicated in the trafficking of the MCTs 1–4 to the plasma membrane (86–88). Recently, it was discovered that the TMPRSS11B protease also regulates the function of MCT4 mediated by CD147 in cancer cells (89).

Over-expression of lactate transporters is a common feature of some cancers with high metabolic rate (90). For instance, high expression of MCT1, MCT4 and its chaperone CD147 is associated with decreased progression-free survival in clear cell renal cell carcinoma, head and neck cancers and neuroblastoma (91–93). In human bladder cancer, high MCT1 expression was associated to shorter overall survival than those with low-MCT1 expression, and the knockdown of MCT1 inhibits cell proliferation, migration and invasion in a cellular model (94). In cervical cancer, it has been shown that CD147 expression was higher in squamous and adenocarcinoma tissue than in their non-neoplastic counterparts, and both MCT1 and MCT4 were more frequently expressed in CD147 positive cases (90, 95). This over-expression of MCT1 and 4 was associated with lymph node and distant metastases in melanoma and adenocarcinoma (96, 97). Interestingly, disruption of MCT1 or MCT4 in renal cell carcinoma, pancreatic cancer, breast cancer and prostate cancer has been shown to exert significant antitumor effects both *in vivo* and *in vitro* with increased accumulation of intracellular lactate (98, 99). In MCT4^−^/^−^ and wild type mice with oral cancer, it was observed that mouse tongues from MCT4^−^/^−^ mice developed significantly fewer and less extended invasive lesions than wild type mice indicating an important role for MCT4 in tumor metastasis (100). Additionally, MCT4 was detected in foci of the basal layer undergoing transformation, in areas of carcinoma *in situ*, and also in invasive carcinomas (100). These findings support the important role of MCTs in tumor metastasis development.

## Lactate in Tumor Microenvironment: Tumor-Associated Acidity

Lactate is largely produced within the TME and is used as an energy-rich substrate, signaling molecule and as an important immune suppressor by tumors (101). TME consist of malignant cancer cells, endothelial cells, cancer associated fibroblasts (CAFs), immune cells and non-cancer cell stroma conformed by numerous peptide factors (growth factors, chemokines, cytokines and antibodies) (20). The glycolytic cancer cells and CAFs are the main producers of lactate, simply because they are the most abundant populations within the neoplasm (101). TME enforce to metabolic adaptability, physical pressure, oxidative stress, nutrient deprivation and competition, immune surveillance as well as adaptability to hypoxic and acidic environment having an enormous impact on tumor malignancy (102). In agreement with this, it was demonstrated that pH 6–6.5 in tumor microenvironment is associated with metastasis, angiogenesis and therapy resistance, a characteristic phenotype of more aggressive tumors (103, 104). This tumor acidification is a consequence of high lactate production in a poorly perfused environment, as well as a high activity of the CAIX (105). CAIX is a transmembrane protein belonging to the α carbonic anhydrase family of zinc metalloenzymes that catalyze the reversible hydration of carbon dioxide to bicarbonate ions and H^+^ (106) that is overexpressed in tumors and is associated with unfavorable responses to first-line therapy (107). The tumor hypoxia induces the expression of the CA9 gene in a HIF 1-α dependent manner; on the other hand it was shown that lactate promotes normoxic expression of CA9 genes through HIF 1-α stabilization independently of hypoxia (108). CAIX acts as an extracellular pH-stat, maintaining an acidic tumor extracellular pH favoring invasion and metastasis (105).

By cooperating with anion exchanger 2 (AE2) and Na^+^/bicarbonate co-transporter 1 (NBCe1), CAIX serves as a pH regulatory component that provides acid-base balance. Interestingly, it was shown that CAIX work in support with diverse acid extruders such as MCT1 and MCT4 (109, 110), and as mentioned earlier, MCT-mediated H^+^ efflux exacerbates extracellular acidification and supports the formation of a hostile environment where cancer cells, that have adapted to these conditions, can outcompete normal cells, which further enhances tumor progression.

The proteoglycan like (PG) domain of CAIX could function as a “proton antenna” to facilitate MCT1 and MCT4 transport activity in hypoxic cancer cells (111). Recently, it was found that CAIV also facilitate the activity of MCT1, MCT2, and MCT4 via a non-catalytic mechanism and requires direct biding between CAIV in the amino acid residue His-88 and a charged amino acid in the extracellular domain of the chaperones CD147 and GP70 (112).

## Lactate Exchange Between Cancer Cells: Metabolic Symbiosis

**Figure 2:**
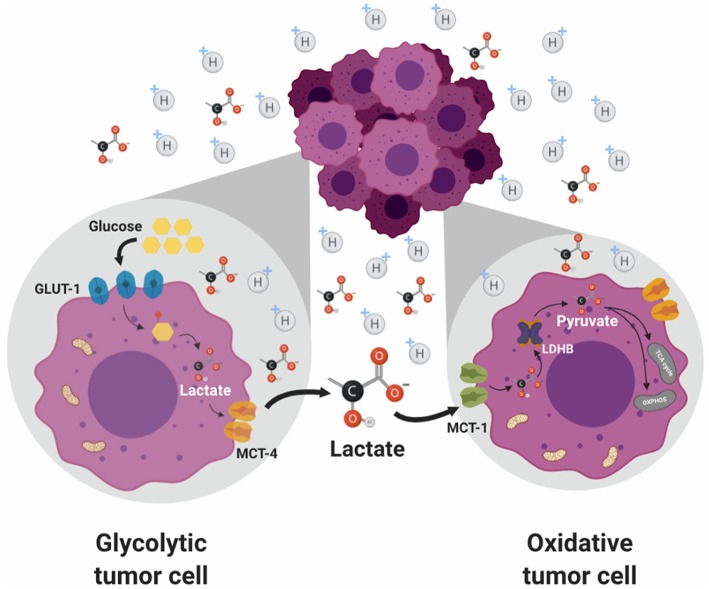
Metabolic symbiosis. Solid tumors are characterized by metabolic heterogeneity. Glycolytic tumor cancers are sustained by a favorable location with high nutritional availability. This phenotype is regulated by a differential expression of MCTs, where glycolytic cells preferentially express MCT4 favoring lactate export. Meanwhile, oxidative cells express MCT1 transporter which preferentially promotes lactate import. Then, lactate is used by these cells as an energetic source due to its conversion to pyruvate which enters the TCA cycle in the mitochondria. The presence of lactate allows a metabolic symbiosis between hypoxic cancer cells (glycolytic) and with normoxic cancer cells (oxidative).

As previously described, the lactate shuttle theory occurs in normal physiology (29). Interestingly, this mechanism is also observed in cancer cells where it is known as “metabolic symbiosis.” Sonveaux et al. (53) found that cervical cancer-derived SiHa cells, which expressed higher levels of MCT1 but lower levels of MCT4, consumed significantly more lactate and less glucose than colorectal cancer WiDr cells, conversely, WiDr cells, which expressed higher levels of MCT4 and lower levels of MCT1, consumed less lactate and more glucose than SiHa cells. Consistent with the proposed “tumor metabolic symbiosis,” metabolically heterogeneous regions within and between tumors were identified, which are regulated by TME conditions such as hypoxia in the center of the tumor and better oxygenated regions in the periphery (113) (Figure 2). Furthermore, it was shown that a high lactate uptake occur only in aerobic tumor regions in breast cancer (34). The oxygenated cancer cells, close to blood vessels, are sustained by a favorable location with high nutritional availability and can establish a metabolic symbiosis with hypoxic cancer cells, essential for the progression of a fast-growing tumor characterized by hypoxic regions. This tumor metabolic symbiosis is supported by differences in MCT1 and MTC4 expression and activity. MCT1 (SLC16A1) is ubiquitously expressed and has a high affinity for lactate (3–6 mM); this transporter is the main lactate exporter where intracellular lactate levels are low (84). On the other hand, MCT4 (SLC16A3) is expressed strongly in glycolytic tissue (114) and has low affinity for lactate (25–30 mM) and it does not import serum lactate (<2 mM) (115). In tumors, it was demonstrated that both, the glucose transporter 1 (GLUT1) and MCT4 were induced in distal hypoxic cells in a HIF 1-α-dependent fashion (116). Instead, tumor cells proximal to blood vessels, expressed the lactate transporter MCT1. These differences in the regulation of the expression and activity of lactate transporters underpin the metabolic symbiotic model.

Despite the avidity by which tumor cells uptake glucose, glutamine or lactate *in vivo*, encounter conditions of nutrient scarcity are often an issue as a result of the increased rate of nutrients consumption and the inadequacies of the tumor vascular supply, for this reason tumors have develop various nutrient scavenging strategies to bypass these limitations, for instance lactate exchange (117).

## Lactate Exchange Between Cancer Cells and CAFs: Reverse Warburg Effect

**Figure 3:**
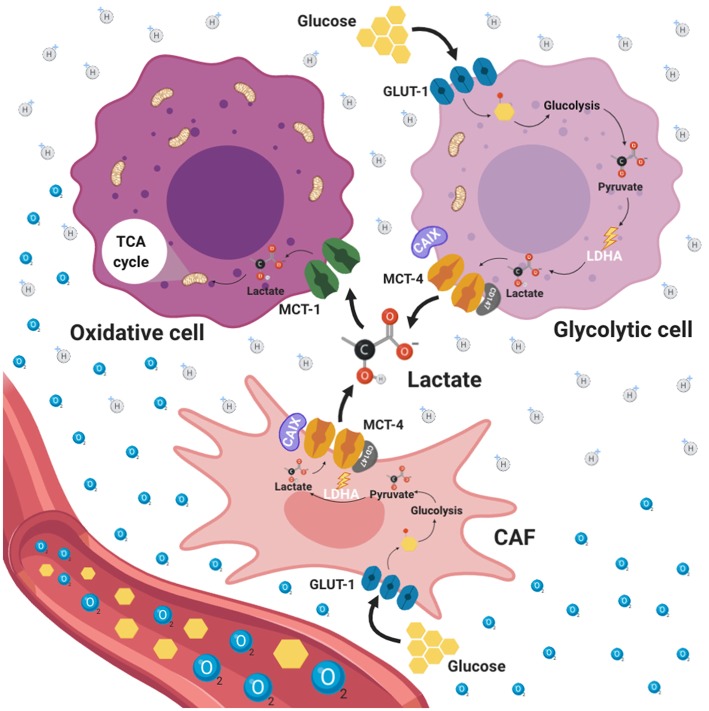
Reverse Warburg effect. Tumor microenvironment (TME) is an ultrastructure consisting of different cell types including tumor cells, stromal cells, immune cells, blood vessels and cellular metabolites such as lactate. TME promotes different processes aimed to enforce metabolic adaptability, oxidative stress, nutrient competition, immune surveillance. This adaptability to hypoxic and acidic environments stimulates tumor malignancy. Tumor cells and cancer associated fibroblasts (CAFs) with a glycolytic phenotype represent the principal source of lactate production within TME which is favored by the presence of GLUT1. Additionally, CAFs exhibit high expression of MCT4 dedicated to lactate export. In this way, CAFs can interchange lactate with oxidative tumor cells which use lactate as a fuel through the TCA cycle. This phenomenon is known as reverse Warburg effect.

Another way by which it is believed that cancer cells survive under nutrient scarcity is by a cross talk between stroma cells from tumor microenvironment and tumoral cells, process known as “reverse Warburg effect,” in which aerobic glycolysis takes place in CAFs, rather than in epithelial cancer cells, fueling cancer cells via metabolite transfer, particularly lactate (118) (Figure 3). CAFs constitute the more abundant cell population in tumors and have been associated with tumor progression, invasion and metastasis directly through paracrine pathways (119). Fibroblasts possess a metabolic phenotype characterized by increased glycolysis and decreased OXPHOS. During tumor initiation, neoplastic cells recruit CAFs to the surrounding area through the ROS production inducing oxidative stress. As a consequence, CAFs suffer DNA damage, initiating several catabolic pathways, such as autophagy and more specifically mitophagy (120). Autophagy is a catabolic process of macromolecules (proteins, lipids) and organelles whereby intracellular components are enveloped in double-membrane vesicles, known as autophagosomes, which ultimately fuse with lysosomes where the content is degraded and recycled into the cytosol (121). On the other hand, mitophagy is a specific process performed in CAFs used for the removal of mitochondria through autophagy. CAFs with dysfunctional mitochondria shift their metabolism toward glycolysis, producing energy-rich molecules, such as lactate, which is exported to the tumor microenvironment and consequently can be used by neighboring cancer cells via oxidative mitochondrial metabolism providing an alternative energy source promoting tumor initiation, progression and metastasis (122). Interestingly, it has been shown that CAFs are able to stimulate cancer cell proliferation and progression through multiple mechanisms. For instance, in a lung cancer model, CAFs underwent increased aerobic glycolysis and promoted the epithelial mesenchymal transition, migration and invasion of non-small-cell lung carcinoma (NSCLC) cells, in contrast, NSCLC cells experienced enhanced oxidative phosphorylation upon CAF stimulation, with an increase in ATP generation, thereby an activation of the PIK3/Akt and MAPK/ERK pathways occurred (123). Furthermore, colorectal cancer cells induce oxidative stress in microenvironment fibroblast, which then undergo metabolic changes, including increased expression of glycolytic enzymes, reduced TCA cycle enzymes and autophagy proteins such as microtubule-associated protein 1A/1B-light chain 3 (LC3), Bcl-2 interacting protein 3(BNIP3), and p62 (124). In this model, the increased autophagy promoted survival of cancer cells by providing nutrients for cell proliferation and protection against oxidative damage. Moreover, hypoxia-induced oxidized ATM promoted the glycolytic activity of CAFs by phosphorylating GLUT1 at S490 and in consequence induced its membrane translocation (125). In addition, the PKM2 expression in CAFs was up regulated by the activation of ATM through PI3K/Akt signaling pathway.

Another study showed that intercellular contact activated stromal fibroblasts, triggering the expression of GLUT1, lactate production, and extrusion of lactate by the *de novo* expressed MCT4 (126). Conversely, prostate cancer cells, upon contact with CAFs, were reprogrammed toward aerobic metabolism, with a decrease in GLUT1 expression and an increase in lactate upload via MCT1. Metabolic reprogramming of both stromal and cancer cells was under strict control of the HIF 1-α, which drove redox-and SIRT3-dependent stabilization of HIF 1-α in normoxic conditions. Prostate cancer cells gradually became independent of glucose consumption, while developing a dependence on lactate driving anabolic pathways and thereby cell growth (126). Lactate shuttle between CAFs (released by MCT4) and tumor cells (absorbed via MCT1) may accelerate tumor cell invasion by activation of TGF-β1/p38 MAPK/MMP2/9 signaling (125).

This reverse Warburg effect provides tumoral metabolic plasticity that enables tumor cells to adapt to variations in microenvironment and represents a change to the paradigm on the metabolism of neoplastic cells, indicating that not all tumors depend on glycolysis (Warburg effect), since some tumors exhibit high dependence of OXPHOS and consequently of mitochondrial function (127). It has been observed that mitochondrial metabolism is important for cancer development. Interestingly, frozen sections of human breast tumors exhibit have a high expression and activity of cytochrome C oxidase (COX), NADH and succinate dehydrogenase in comparison to normal cells (122). This effect was related to greater aggressiveness of the tumors. Moreover, it was shown that the mitochondrial complex I NADH dehydrogenase activity is a critical player in the aggressive phenotype in breast cancer through the regulation of NAD^+^/NADH redox balance, mTORC1 activity and autophagy (46). The mitochondrial function and its relation with cancer development is a very interesting topic excellently discussed in the review of Vyas et al. (5).

## Lactate as a Key Molecule in Regulation of the Immune Response in Cancer

The immune system is responsible for protecting the body from damage caused either by pathogens or by tumor cells through the detection and elimination of aberrant cells (128). The presence of neoplastic cells causes the activation of both, the innate and adaptive immune responses in order to maintain homeostasis (129). Nevertheless, tumor cells have developed different mechanisms to evade the immune system including a constant remodeling at the genetic, epigenetic and metabolic levels, in order to resist apoptosis and select tumor variant cells resistant to immune recognition. In addition, TME favors the induction and recruitment of different immune cells and molecules constituting an immunosuppressive environment, favoring the development of the tumor mass (130). It has been shown that metabolic alterations play an important role in cancer development, progression and maintenance (131). As part of the high metabolic rate and reprograming, tumor cells secrete metabolic products as lactate, which is thought to act as an important oncometabolite in the metabolic reprogramming of cancer. In turn, the high levels of secreted lactate promote acidosis in the tumoral environment favoring processes such as metastasis, angiogenesis and importantly, immunosuppression, which has been associated to a worse clinical prognosis (132).

Different cells are involved in the recognition and elimination of tumor cells including natural killer (NK), natural killer T (NKT) cells, macrophages, dendritic cells (DC), macrophages, and lymphocytes (129).

NK cells induce the destruction of stressed cells, cells infected by viruses or bacteria as well as tumor cells (133); this last action performed through their “killer” receptor (KIR) (134). The major histocompatibility class I (MHC-I) complex is recognized by KIR receptors inhibiting the activation of NK cells, however, tumor cells display diminished amounts of MHC-I which in consequence triggers the activation of NK cells, with the subsequent release of their cytoplasmic granules containing granzyme and perforin finally inducing cell lysis (135). Nevertheless, tumor cells inhibit the activation of NK cells triggered by lacking of MCH class I expression through the release of soluble molecules such as MHC class I chain-related protein A (MICA) and MHC class I chain-related protein B (MICB), which bind to the activator receptor (NKG2D) on NK cells surface causing the endocytosis and its subsequent degradation, leading to the inactivation of NK cells (136). In addition to this mechanism for inhibiting NK cells action, it has been demonstrated that the presence of lactate induces the apoptosis of NK cell, since lactate decreases the intracellular pH resulting in mitochondrial dysfunction in colon cancer-derived cells (137). It was also observed that those NK cells that migrate to the tumor cannot regulate intracellular pH, causing mitochondrial stress and subsequent apoptosis.

Using a pancreatic cancer-derived mouse xenografted model, the silencing of LDH caused a reduction of the tumor associated to a better cytolytic activity of NK cells (138). Thus, tumor microenvironment easily affects immune actions by producing lactate, favoring the development of cancer.

**Figure 4:**
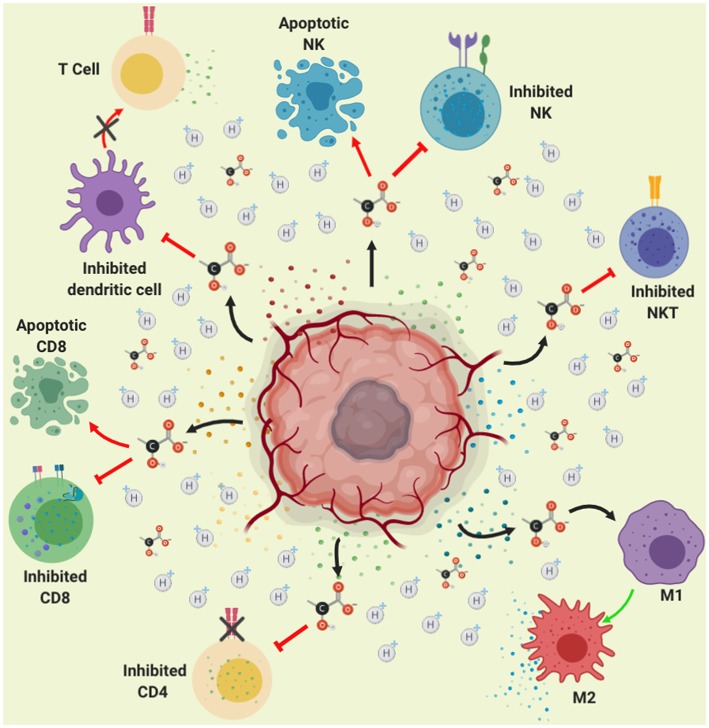
Role of lactate in immune suppression. Lactate secretion by tumor cells promotes acidification of the tumor microenvironment (represented in yellow color). This acidification of the medium, reduces the pH within the immune cells affecting signaling pathways finally causing inhibition of the activation and proliferation of CD4, CD8, NK, NKT, and dendritic cells. Moreover, lactate-induced acidification causes apoptosis in CD8 lymphocytes and NK cells, thus lactate contributes to immune evasion. Furthermore, the acidification of the medium causes the polarization of the macrophages toward the M2 subpopulation, which favors growth, invasion and migration of the tumor.

NKT are another immune cells with antitumor activity, which recognize glycolipids through the CD1d receptor on the tumor cells, this interaction activate its antitumor action releasing the content of their cytoplasmic granules (perforin and granzyme B) as well as cytokines favoring activation of both, innate and adaptative immune response (139). It has been shown that lactate present in TME, blocks IFNγ, and IL-4 production from NKT cells, since lactate inhibits mTOR signaling due to the inhibition of the nuclear translocation of promyelocytic leukemia zinc-finger (PLZF) avoiding the activation of NKT cells (140). These results are in agreement with those reported recently by Kumar et al. (141), showing that high lactate environment is detrimental for NKT cell survival and proliferation. This indicates that the production of lactate by the tumor microenvironment inhibits the anti-tumor action of NK and NKT cells, promoting tumor development (Figure 4).

DCs are antigen-presenting cells, which play a major role in the innate and adaptive immune responses (142). DCs main function relies on its ability to detect and phagocytize pathogens, but also on the recognition of tumor cells, by processing and presenting antigens that finally activates virgin T lymphocytes (143). DCs present the antigen through the MHC-II activating CD4^+^ T (LT) or helper cells, that depending on the produced cytokine environment, these will differentiate into a variety of subpopulations of helper T cells mainly Th1, due to the action of IL-12 and IFN-γ produced by NK, NKT and macrophage cells (144, 145). In addition, DCs present antigens to cytotoxic T lymphocytes (CTL) or CD8, these cells recognize tumor antigen through MHC-I producing the elimination of tumors (146). After the recognition of non-own peptides (such as tumor antigens) through MHC-I, CD8 T cells will be activated causing cytokine release, mainly TNF-α and IFN-γ (147), as well as their cytoplasmic granule (perforin and granzime B) content toward neoplastic cells, it also induces apoptosis through the interaction of dead molecules such as Fas with Fas Ligand present in the tumor cells, causing lysis and apoptosis of the tumor cells (148). The T lymphocytes (CD4 and CD8) play an important role in eliminating tumor cells; this process is known as immune-surveillance (149). The lack of response of the lymphocytes could also be due to the fact that it has been shown that lactate affects the dendritic cells, prevents their differentiation and makes them tolerogenic, leading to an increase in the production of IL-10, a potent immuno-suppressive cytokine (150).

IL-10 is an anti-inflammatory cytokine with potent immune-suppressive action, since it inhibits the production of pro-inflammatory cytokines such as IFNγ, TNFα, IL-1β, and IL-6; moreover, IL-10 prevents DC maturation in part by inhibiting the expression of IL-12, necessary for Activating of type 1 helper T cells and stimulates the production of cytotoxic T cells and NK cells. It also stimulates the production of interferon (151, 152). Also, IL-10 inhibits Th1 differentiation and production of IL-12 (153). In addition, IL-10 inhibits the expression of MCH-I and different co-stimulatory molecules inhibiting T cell activation (154, 155). Several reports indicate an IL-10 increase in serum levels in patients with different types of cancer such as hepatocellular, head and neck, lymphoma, leukemia and melanoma (155). Therefore, over regulation of IL-10 production by tumor cells promotes tumor progression through the escape of immunosurveillance performed by NK, CD4, and CD8 lymphocytes. In addition, lactate produced by the tumor cell promotes the overexpression of IL-23, present in different types of tumors (colon, breast, stomach, melanoma) (156), the presence of this cytokine promotes expression of IL-17, Matrix metalloprotease 9 (MMP-9), increases angiogenesis and reduces the infiltration of CD8 in the tumor, promoting immunosuppression and tumor growth (157). Thus, increased IL-10 favors tumor microenvironment.

A study quantified lactate levels in the serum of patients with different malignancies (lymphoid malignancies, myeloid malignancies, breast cancer, gastrointestinal cancer, urogenital cancer, sarcoma, lung cancer, melanoma and other types of cancer), finding high levels of lactate, furthermore, the authors demonstrated that lactate inhibits T-cell proliferation and alters the cytokine production of CTLs in cultured CTLs (158), therefore, lactate promotes immunosuppression and the development of cancer through the inhibition of T lymphocytes.

Lactate produced by tumor microenvironment participates in immune escape through an inhibition of lymphocytes activity. As demonstrated in samples of melanoma patients showing that high LDHA expression is associated not only with poor prognosis, reduced disease-free survival, but also with lower expression of T cell markers (159). Moreover, in a melanoma mouse model it was found that tumor-derived lactate reduced the numbers and activity of CD8^+^ T cells as well as NK cells, both *in vitro* and *in vivo*. This because lactate concentrations above 20 mM caused the apoptosis of T and NK cells, which may explain smaller proportions of T cells and NK cells in tumors with higher concentrations of lactate (159). Similar results were observed recently by Daneshmandi et al. (160), where blocking of LDHA in melanoma tumors effectively enhances infiltration of CD8^+^ T cells and NK cells in the tumor microenvironment. Interestingly, they also demonstrated that blocking LDHA in tumor cells improves the efficacy of anti-programmed cell death-1 (PD-1) therapy in melanoma (160). Therefore, this is a mechanism used by tumor cells to evade the action of T lymphocytes. Moreover, the acidic pH (6.5) suppress T-cell functions including IL-2 secretion and the activation of T-cell receptors and the treatment with proton pump inhibitor (esomeprazole) delayed cancer progression in tumor bearing mice (161). Recently, it was shown that the acidic pH environment (6.6) blockades the T-cell activation and decreases IFNγ secretion (162).

There is great evidence indicating that lactate promotes immunosuppression thus preventing the recognition of tumor cells and favoring carcinogenesis. Interestingly, lactate has a different effect on macrophages, as demonstrated by Colegio et al. (163) tumor-cell-derived lactate has an important impact in the macrophages polarization and the promotion of tumor growth. This is because lactate induces mainly vascular endothelial growth factor (VEGF) and arginase 1 (Arg1) expression via HIF 1-α, favoring the TAM polarization. Moreover, the upregulation of VEGF and Arg1 in macrophages contributes to the development of cancer since tumor growth is supported by inducing neovascularization and by providing the substrates for cancer cell proliferation. Otherwise, it has been demonstrated that lactate activate human macrophages to a M2 phenotype and stimulate the secretion of Chemokine (C-C motif) ligand 5 (CCL5) by activation of Notch signaling in macrophages. The authors also found that CCL5 increased cell migration and induced cancer cell epithelial to mesenchymal transition in a breast cancer cell model (164). Additionally, it was shown that lactate activates the ERK/STAT3 signaling inducing the M2 macrophage polarization favoring proliferation, migration, and angiogenesis in a breast cancer model (165).

## Lactate in Tumor Metastasis and Therapy Resistance

Metastatic dissemination represents a malignant character of cancer with important clinical consequences since the majority of cancer-associated deaths are caused by metastatic disease rather that the primary tumors (166). Intratumor lactate levels can be used as a prognostic factor and a therapy response biomarker. It has been shown that high concentrations of lactate in biopsies of cervical, lung, head and neck, colorectal and breast cancers are associated with an increased risk for developing metastasis, and such levels of lactate indicate a bad prognosis for survival in cancer patients (30, 31, 35, 167) (Table 1). In human astrocytomas, a positive correlation between the grade of lesion and high lactate concentration was found using stereotactic brain biopsy specimens (36). Moreover, using imaging bioluminescence from primary cryo-tumor sections of human cancers, lactate concentrations were significantly higher in cervical tumors with metastatic spread (30). Another study encompassing 34 biopsies from patients with cancers of the head and neck, it was demonstrated that elevated tumor lactate concentrations are associated with the subsequent development of nodal or distant metastases (31).

The measurement of intratumor lactate levels using non-invasive methodologies; such as nuclear magnetic resonance (MRS) is currently used (168). In HER2-positive breast cancer lactate can be used as a quantitative and non-invasive biomarker of sensitivity to trastuzumab. Using MRS in a cohort of 39 frozen HER2-positive breast cancer specimens of patients who showed response to trastuzumab, a positive correlation between the transcript levels of HER2 and increased intratumor lactate concentration was found, moreover *in vitro* analyses using HER2-high expression (ZR75.30, SKBR3, BT474, and HCC1954) or HER2-low expression (MDAMB361 and MDAMB453) cell lines, it was found a direct correlation between HER2 transcript levels and lactate content in milieu (169).

Resistance to therapy is frequently developed during the clinical application of antineoplastic agents and is a major obstacle in the treatment of malignant cases (170). A substantial percentage of cancer patients exposed to an antineoplastic agent either does not benefit from the treatment (primary resistance) and shown reduced responsiveness or undergo tumor relapse progression (secondary resistance) (171). This resistance may be due to both, cell-autonomous and non-cell-autonomous mechanisms, TME is important in the initiation and maintenance of non-cell-autonomous drug resistance through various mechanisms including hypoxia, extracellular acidity and production of soluble factors such as lactate (172) (Figure 1). The role of lactate in resistance to therapy has been demonstrated using *in vivo* and *in vitro* models. High lactate concentration in xenografted Nude mice with five human HNCSCC cell lines treated with irradiation (4 Gy) within 6 weeks correlates with radio resistance (37). In NSCLC, it has been shown that lactate is a key molecule involved in resistance to therapy based on tyrosine kinase inhibitors (TKIs), specifically with c-MET receptor tyrosine kinase inhibitor JNJ-605 and the epidermal growth factor receptor (EGFR) inhibitor erlotinib (173). In this work, the authors demonstrated that prolonged treatment with these TKIs induced lactate production by tumor cells, which in turn instructed the TME cells to produce hepatocyte growth factor (HGF), enforcing drug resistance and tumor progression. Targeting tumor lactate metabolism was sufficient to overcome resistance, demonstrating the causative role of lactate in resistance to therapy. Another study tested several metabolic inhibitors including BEZ235, GDC0980 (dual PI3K/mTOR inhibitors), or LY294002 and GDC0942 (PI3K inhibitors) showing an inhibition of cell proliferation of breast cancer cells in high glucose media (54). Nevertheless, when lactate was used as the primary metabolic substrate these cells were completely resistant to these inhibitors, suggesting that cancer cells bypass the need for glycolysis by utilizing lactate and are thus less sensitive to PI3K/mTOR inhibitors.

Due to the role of lactate in tumor initiation and metastatic dissemination previously mentioned, impairing lactate homeostasis is a promising approach for cancer therapeutics and has been implemented in several preclinical and clinical trials, it is also essential to establish a synergy between lactate inhibitors and other adjuvant therapies.

## Therapeutic Approaches in Lactate Metabolism

### Targeting Lactate Production

Glycolytic tumors undergo a metabolic reprogramming transforming themselves into a highly glycolytic and poorly oxidative phenotype with lactate formation as the end product despite normoxic conditions. This high glycolytic metabolism supplies precursors for biomolecules in cellular structure and processes allowing cell survival and proliferation (174, 175). In agreement with the above mentioned with regard to essential role of lactate in tumor development, metastasis and its role in drug resistance, impairing the lactate biogenesis could be a promising approach to cancer therapeutics (Table 2).

**Table 2:** Approaches for inhibit lactate production and transport.

**Inhibitor(s)**	**Mechanism of action**	**Type of cancer or cell/animal model**	**Research phase**	**Limitations**	**References**
5 designed peptides (QLYNL, LIYNLL, IYNLLK, KVVYNVA, and KVVYNV)	LDHA tetramerization inhibition, affecting the activity of the enzyme	None	*In silico* modeling	*In vivo* investigation of these peptides on cancer cell lines is needed to evaluate their biological potential	(176)
Compound 24	24c interacts directly with the binding pocket of LDHA affecting the activity of the enzyme	Pancreas carcinoma (MiaPaCa-2)	Pre-clinical	No limitations were shown, indeed 24c did not affect the body weight of the mice, indicating low toxicity of the compound	(177)
1-(Phenylseleno)- 4-(Trifluoromethyl) Benzene (PSTMB)	This allosteric inhibitor of LDHA modifies the pyruvate binding site due to conformational changes on the enzyme by non-competition inhibition	Large cell lung cancer (NCI-H460) Breast cancer (MCF-7) Hepatocellular carcinoma (Hep3B) Malignant melanoma (A375) Colorectal adenocarcinoma (HT29) Murine lung cancer cells (LLC)	Pre-clinical	No limitations were shown, even in normal human bronchial epithelial BEAS-2B cells, the cytotoxic effect of PSTMB was limited	(178)
Oxamate siRNA LDHA gene	Oxamate is a non-competitive inhibitor which has same the structure of pyruvate, this compound inhibits LDHA activity Small interfering RNA use to regulate the expression of LDHA gene	Breast cancer (MCF-7 and T47D)	Pre-clinical	No limitations were shown	(179)
Compounds 5 and 11	Both compounds maintain the same hydrogen bond interactions with LDHA, however 11c has extra interactions which could give rise to its inhibitory activity against LDHA	Osteosarcoma (MG-63)	Pre-clinical	No limitations were shown, however further experiments with different cancer models are needed to ensure its biological efficacy	(180)
OxamateGalloflavin	Oxamate a non-competitive inhibitor hinders LDH activity Galloflavin inhibits human LDH isoforms preferentially binding the free enzyme, without competing with the substrate or cofactor	Liver cancer (PLC/PRF/5)	Pre-clinical	No limitations were shown	(181)
siRNA LDHA geneFX11FK866	Small interfering RNAs for knocking-down the expression of LDHA gene FX11 is a competitive inhibitor of LDHA FK866 hinders the NAD^+^ synthesis through direct inhibition of Nicotinamide Phosphoribosyl transferase (NAMPT)	B-lymphoid cells (P493) Pancreatic cancer (P198)	Pre-clinical	The combination of both compounds was toxic for P493 cells causing a reduction of mitochondrial membrane potential resulting in profound inhibition of cell proliferation In the *in vivo* assay, animals treated only with FX11 did not lose weight or showed any alterations in blood and chemistry studies. However, two of five studied animals treated with FK866 did show mild thrombocytopenia. Remarkably, the combination of FX11 and FK866 increased BUN	(68)
AZD3965	Selective inhibitor of human MCT1 with additional activity against MCT2 This compound hinders lactate transport, consequently increasing intracellular levels followed by glycolytic feedback and increased flux into the TCA cycle	Human diffuse large B-cell lymphomas (HBL-1 and TMD8) Human B-cell lymphoma (WSU-DLCL-2 and SU-DHL10) Lymphoblast (HT) B-cell non-Hodgkin lymphoma (Karpas-422 NHL) Raji Burkitt's lymphoma cells	Pre-clinical	This potent inhibitor of MCT1 showed a reduction in growth of different cell lines especially hematological types. Although the inhibitory effect, some types of cancers express both transporters MCT1 and MCT4, in this regard MCT4 may be continuing the lactate transport suggesting a resistance to the monotherapy	(182)
AR-C155858	Selective monocarboxylate transporter (MCT1 and MCT2) which affects lactate uptake in a time dependent manner with slow reversible features	Murine breast cancer cell line, 4T1	Pre-clinical	No limitations were shown	(183)
CHC (α-cyano-4-hydroxycinnamic acid)DIDS (4,4′-diisothiocyanatostilbene-2,2′-disulphonic acid) Quercetin	CHC inhibits different MCT isoforms, namely MCT1 as a primary target. This compound interacts with the outside proteins of the membrane affecting lactate efflux, consequently arresting glycolysis DIDS is a MCT1 inhibitor, the interaction between one of the isothiocyanate groups of DIDS with a lysine residue of MCT1 could affect the transporter activity Quercetin is a MCT inhibitor, specifically MCT1 and MCT2, the lactate and proton transport promotes intracellular acidification	Colorectal cancer cells (HCT15 and RKO)	Pre-clinical	Tested compounds are wide MCT inhibitors	(184)
BAY-8002	Selective inhibition MCT1 which potently suppress bidirectional lactate transport	Hematopoietic malignancies, Raji, and Daudi Burkitt lymphoma cells	Pre-clinical	A limited antitumor efficacy was observed in the *in vivo* models suggesting a limited effect of the MCT1 blockage. Thus, cells exhibit a capability to adapt to long-term inhibition of MCT1 Only a small proportion of cell lines tested showed a significant reduction of cell viability indicating the necessity for testing MCT1 in clinical tests	(185)
Syrosingopine	Increases intracellular lactate due the inhibition of both MCT transporters (MCT1 and MCT4)	HeLa, HAP1, HL60 cells, liver tumor mouse model	Pre-clinical	No limitations were shown	(186)

Several methods have focused in targeting lactate production, for instance Le et al. (68) demonstrated an increase in oxygen consumption, ROS production and late cell death even necrosis, in P493 cells (B-lymphoid cells) after inhibiting the expression of LDHA using siRNAs as well as employing a Gossypol analog as FX11, a direct competitive inhibitor of LDHA. Another compound FK866, that hinders the NAD^+^ synthesis through direct inhibition of Nicotinamide Phosphoribosyl transferase (NAMPT) was used. The use of both molecules was toxic for P493 cells either alone or in combination, causing a reduction of mitochondrial membrane potential resulting in profound inhibition of cell proliferation. Tumor xenograft models using P493 (lymphoma) and P198 (pancreatic) cells were performed in order to demonstrate the potential of both compounds in the inhibition of tumorigenesis *in vivo*. FX11 effectively inhibited tumor growth in xenografts derived from both cell lines; the combination of FX11 with FK866 induced tumor regression in the human lymphoma xenograft model. These results showed that LDHA is required for tumor progression where targeting cancer metabolism using small molecules provides a manner for controlling tumor growth.

As part of the responses of tumor metabolic stress, heat shock proteins (HSPs) are rapidly expressed, stress signals include a wide variety of physiological and environmental insults, which are proven to be essential for survival, this protective mechanism is usually referred as “Heat shock response” (HSR). Moreover, there is evidence that a wide range of human cancers exhibit an over-expression of HSPs providing a meaning for cell proliferation, differentiation, invasion, metastasis and evasion of the immune system (187). The HSF-1 transcriptional factor regulates the expression of HSPs but also regulates glucose metabolism by activating the expression of LDHA (188). In order to set the connection between HSR and LDH, an inhibitor of the LDH activity (Oxamate) by direct competition with its natural substrate was used in a hepatocellular carcinoma (HCC) derived cell model. Oxamate, was found to impact the constitutively activated HSR by reducing the levels of the HSP-27,−72, and−90 (181). Additionally, Galloflavin, hindered the ATPase activity of HSP 72 and 90, both compounds resulted in cell senescence. Taken together, the inhibition of LDH could be an efficient way to reduce the constitutively activated HSR in cancer cells by hindering the function of the three major molecular chaperones involved in tumorigenesis.

Targeting lactate metabolism as a therapeutic approach to defeat drug resistance has also been tested in different tumors. For instance, Das et al. (179) induced tamoxifen-resistant breast cancer cell lines (TAM-MCF-7 and -T47D) in order to establish a connection between LDHA and the induced pro-survival mechanism autophagy. The pharmacological and genetic inhibition of LDHA re-sensitized the TAM-resistant breast cancer cells to tamoxifen, but also inhibited the autophagy process therefore increasing cell death. These results provided a link between LDHA and Beclin-1, an important regulator of autophagy, in the induction of the cytoprotective autophagy of TAM-resistant breast cancer cells. Moreover, the depletion of LDHA reverted the EMT-like process attenuating the invasive and migratory properties of TAM-resistant cells. These results reveal that targeting the LDHA enzyme may be a novel strategy to combat glycolytic chemo-resistant cancers.

Given the importance of lactate metabolism in different types of cancers, the discovery and development of new molecules that could inhibit LDHA activity is urgently needed. Only a few molecules have started tests in clinical trials, for this reason there is a trend to optimize existing compounds, such is the case of compound 5, that was used as a template for molecular docking, then the 200 top-ranked compounds with the highest total binding scores were selected, however, only 1 molecule (compound 11: 11c) from 7 candidates was employed for further biological validation (180). 11c maintains the same hydrogen bond interactions as compound 5 in the binding model and exhibits extra hydrogen bond interactions with the residues Asp 194 and Thr 247 in LDHA, which could give rise to its inhibitory activity against LDHA. The *in vitro* assays reveal the potential action of 11c in the metabolism of an osteosarcoma-derived cell line, MG-63. These cells exhibit a dose-response effect to 11c, where lactate formation significantly diminished with the subsequent extracellular acidification rate (ECAR) decrement, consistent with a poor lactate synthesis. In addition, the use of 11c upregulated the oxygen consumption rate (OCR) indicating a metabolic switch from lactate production to pyruvate consumption. In relation to cell proliferation, 11c promoted apoptosis in the same dose dependent manner, thus impacting cell proliferation. Taking together, compound 11 is a new potent LDHA inhibitor, demonstrated by its ability to induce the reprogramming of MG-63 cancer cells metabolism from glycolysis to mitochondrial respiration decreasing cell survival. Nevertheless, further experiments using different types of cancers are needed to ensure its biological efficacy.

As for the optimization of small molecules, compound 24c is a novel potent LDHA inhibitor obtained by a hit-to-lead optimization from an in-house library. 24c interacts directly into the binding pocket of LDHA, forming a direct hydrogen bond interaction with Asn137, Arg168, His192, and Gln99 of the enzyme causing a metabolic alteration by enhancing oxidative phosphorylation and reducing lactate formation in cancer cells, which might contribute to their anti-proliferation effect. In addition, this compound showed a reduction of cell growth as well as apoptosis and cell cycle arrest in a dose dependent manner against MiaPaCa-2 cells derived from pancreas carcinoma. Furthermore, 24c suppressed the tumor growth in the xenograft model. Additionally, the evaluation of the metabolic profile in MiaPaCa-2 cells treated by 24c exhibited a decreasing in ECAR and lactate production but an increased OCR value. Consequently, these observations suggested that 24c could be used as a lead pharmacophore for the development of new potent LDHA inhibitor (177).

Through a screening of novel inhibitors, Kim et al. (178) found several promising selenobenzene compounds with inhibitory effects on LDHA activity. The most potent inhibitor of the activity of LDHA was 1-(phenylseleno)- 4-(trifluoromethyl) benzene (PSTMB), this compound acts as an allosteric inhibitor modifying the active site where pyruvate binds, trough conformational changes that lead to the inhibition of enzymatic activity. Experimental assays indicated that PSTMB inhibited cell proliferation in several tumor cell lines including lung cancer (NCI-H460), breast cancer (MCF-7), hepatocellular carcinoma (Hep3B), malignant melanoma (A375), colorectal adenocarcinoma (HT29) and murine lung cancer cells (LLC). Furthermore, PSTMB incremented ROS generation and reduced the stability of the mitochondria inducing intrinsic pathway-mediated apoptosis of cancer cells. Additionally, LDHA activity and lactate production were clearly reduced by PSTMB under hypoxic and normoxic conditions, this suppression was mainly mediated by the inhibition of the enzyme activity, and not by the regulation of its expression. Summarizing, this novel selenobenzene, PSTMB, was found to be a potent inhibitor of the human LDHA enzyme.

Recently, peptides have been used as new class of drugs for the treatment of different diseases including cancer (189–191). Owing to the protein-protein interaction (PPI), peptides have been used as a novel and powerful tool in drug discovery. Recently, novel peptides aiming to disrupt the subunit association of LDHA during its tetramerization process have been designed through *in silico* methods, designed to impact the activity of the enzyme. These peptides were developed based on its active conformation and the interaction interface of LDHA subunits where the N-terminal arm (residues 5–17) acts as an anchor to maintain the position and distance between the two LDHA subunits. Thus, these new peptides mimic the anchoring of the LDHA subunits avoiding its tetramerization (176). These novel anti-cancer agents designed for therapy have promising advantages like low toxicity, ease of synthesis and high target specificity whereas the classical pharmalogical therapeutics. However, *in vivo* investigation of these peptides and its effects on cancer cell lines is needed to evaluate the biological potential.

### Targeting MCTs

The inhibition of the MCTs has also been implemented as a therapeutic strategy. Although there are only few reports inhibiting specifically the MCT, these have showed promising results in different neoplasms.

The AR-C117977 was first identified as an immunomodulatory compound with antiproliferative properties on T lymphocytes, where the MCT1 was identified as its target (192). The AZD3965 is a derivate compound from AR-C117977 with potent inhibition of the MCT1 with additional activity against MCT2. The main action of the compound is the inhibition of lactate transport inducing an acute increase in intracellular lactate levels followed by glycolytic feedback and increased flux into the TCA cycle (182). This compound inhibited the proliferation of several lymphoma cell lines. Even, the combination of AZD3965 with other compounds like inhibitors of GLS1, doxorubicin or rituximab resulted in enhanced inhibition of cell growth and increased cell death in the tested cell lines (182). Nevertheless, the status of MCTs could be contributing to the observed inhibitory effect, thus the evaluation of MCTs expression in the tested models could provide better insights for inhibitory molecules.

The effect of AZD3965 was tested along with AR-C155858 in a murine breast cancer-derived cell line, 4T1 (183). The authors found that both compounds exhibited a time-dependent inhibition of lactate uptake and very importantly, this inhibition was slowly reversible, indicating that such effect could offer potential benefits in cancer treatment. Likewise, Amorim et al. (184) tested the antiproliferative effects of three compounds in colorectal cancer derived cells finding that colorectal cancer cells, HCT15 and RKO decreased its glycolytic rate and enhanced cell death in the presence of any of the probed molecules. Interestingly, the cytotoxic effect exerted by 5 fluoro-uracil was potentiated when using together with those drugs. Moreover, targeting MCT1 and MCT4 with syrosingopine in cell models from different cancer types, Bejamin et al. (186) found an increase in intracellular lactate in HeLa cells, and the liver mouse model showed reduction in lactate concentration in nodules after syrosingopine treatment. Importantly, an increase in lactate levels in serum from syrosingopine-treated mice and a synergistic effect to metformin anti-properties was shown.

A high throughput examination of over 3 million compounds measuring lactate import-dependent intracellular acidification identified BAY-8002 as a potential MCT1 inhibitor (185). Then, authors showed the antiproliferative properties in different cells lines, where Daudi and Raji cells were most affected by this compound. The *in vivo* testing of BAY-8002 determined its capacity to decrease tumor mass over time using different concentrations ranging from 40 to 160 mg/kg, without affecting body weight. Importantly, chronically exposed cells developed resistance to MCT1 inhibition probably due to the increment of MCT2 and MCT4 expression in resistant cells indicating that different molecular mechanisms could be involved in treatment resistance.

## Concluding Remarks

Lactate is not only considered as a waste product derived from fermentative cell metabolism, but instead is a powerful molecule that contributes to both, the onset and progression of cancer, favoring metastasis and tumor angiogenesis. In tumor microenvironment, lactate establishes metabolic coupling between cancer cells, immune cells and stromal cells, acting as an interchangeable metabolite in the tumor mass. Oxygen availability defines different metabolic phenotypes because their location within the tumor, where hypoxic central areas display a higher lactate concentration. Thus, the development of new tools for quantifying intra-tumoral molecules to trace lactate accumulation and consumption by tumors represents a huge challenge in cancer research.

Lactate participates also in the immune escape through the inhibition of lymphocytes activity and induces the M2 macrophage polarization associated to tumor progression. In addition, it is currently known that metabolic plasticity exhibited by tumors allows the development of treatment resistance mechanisms due to adaptation to metabolic changes, impacting the effect of anti-metabolic drugs. Thus, lactate-induced resistance to therapy represents the major obstacle in the elimination of malignant tumors. For this reason, it is necessary to pursuit for more studies aimed to determine synergistic combinations including lactate dehydrogenase and MCTs inhibitors for developing reliable and effective treatments in cancer.

## Author Contributions

KC-L and JM-M: conception and design. KC-L, LC-M, DR-H, AG-C, and JM-M: wrote and critically review the manuscript. KC-L, LC-M, DR-H, and JM-M: figure design and elaboration. JM-M: directed manuscript.

### Conflict of Interest

The authors declare that the research was conducted in the absence of any commercial or financial relationships that could be construed as a potential conflict of interest.
